# Moderate Aortic Stenosis—Advanced Imaging, Risk Assessment, and Treatment Strategies

**DOI:** 10.1016/j.shj.2023.100279

**Published:** 2024-02-01

**Authors:** Rik Adrichem, Mark M.P. van den Dorpel, Alexander Hirsch, Marcel L. Geleijnse, Ricardo P.J. Budde, Nicolas M. Van Mieghem

**Affiliations:** a Department of Cardiology, Thoraxcenter, Erasmus University Medical Center, Rotterdam, The Netherlands; b Department of Radiology and Nuclear Medicine, Erasmus University Medical Center, Rotterdam, The Netherlands

**Keywords:** Advanced imaging techniques, Aortic valve interventions, Cardiac damage, Moderate aortic stenosis, Risk assessment

## Abstract

Moderate aortic stenosis is increasingly recognized as a disease entity with poor prognosis. Diagnosis of moderate aortic stenosis may be complemented by laboratory tests and advanced imaging techniques focused at detecting signs of cardiac damage such as increase of cardiac enzymes (N-terminal pro-B-type Natriuretic Peptide, troponin), left ventricular remodeling (hypertrophy, reduced left ventricular ejection fraction), or myocardial fibrosis. Therapy should include guideline-directed optimal medical therapy for heart failure. Patients with signs of cardiac damage may benefit from early intervention, which is the focus of several ongoing randomized controlled trials. As yet, no evidence-based therapy exists to halt the progression of aortic valve calcification.

## Introduction

Aortic stenosis (AS) is the most common valvular heart disease in the adult population and its prevalence is expected to rise because of further aging.^1^^,^^2^ Severe AS affects quality of life and is associated with an impaired prognosis. Symptomatic severe AS is an undisputed indication for aortic valve replacement therapy. Less is known about the clinical relevance of moderate AS.

Observational studies revealed that also moderate AS may shorten life expectancy.^3^^,^^4^ This suggests that aortic valve replacement may possibly improve symptoms and prognosis in selected patients with moderate AS. However, correct identification of patients who may benefit most from intervention is challenging, as moderate AS may have a heterogeneous clinical presentation and progression to severe AS is variable. Furthermore, impaired left ventricular ejection fraction (LVEF) and impaired flow patterns may obscure echocardiographic Doppler measurements and heart failure symptoms may be attributed to the reduced cardiac function rather than to the AS.

Herein, we focus on diagnostic challenges, advanced imaging techniques as well as current and novel treatment strategies for patients with moderate AS.

## Diagnosis of Moderate AS

Echocardiography remains the key diagnostic modality in the evaluation of AS. Easily determinable echocardiographic parameters such as the peak aortic jet velocity, mean pressure gradient (as determined by the simplified Bernoulli equation), and the aortic valve area (AVA; as calculated with the continuity equation) provide valuable prognostic insights.^5^ By convention, moderate AS is defined by a peak aortic valve velocity of 3.0 to 4.0 m/s, mean pressure gradient of 20 to 40 mmHg and AVA of 1.0 to 1.5 cm^2^.^6^^,^^7^ Nevertheless, echocardiographic measurement errors may lead to underestimation of aortic valve gradients, because of 1) nonparallel echo beam orientation in relation to flow-direction, 2) underutilization of echo windows (in particular, right-parasternal and suprasternal views), and 3) underutilization of optimal probes (pencil probe rather than large 3D print-plate). Also, the AVA may be overestimated as a consequence of miscalculation because of LVOT velocity-time integrals measured too close to the aortic valve or overtracing of the Doppler profile. Finally, planimetric measurements of AVA in bicuspid AS are often overestimated because of the dome-shaped orifice and measurement at the wrong level.^8^

Moreover, the above mentioned criteria for moderate AS only hold true in the presence of normal transvalvular flow patterns (i.e., transvalvular flow rate ≥250 mL/s and/or indexed stroke volume >35 mL/m^2^). Low transvalvular flow may generate discordant findings between gradients and AVA, which complicates the overall interpretation of AS severity even further.^9^ Various methods have emerged to resolve inconsistencies with discordant echocardiography findings.

**Figure 1:**
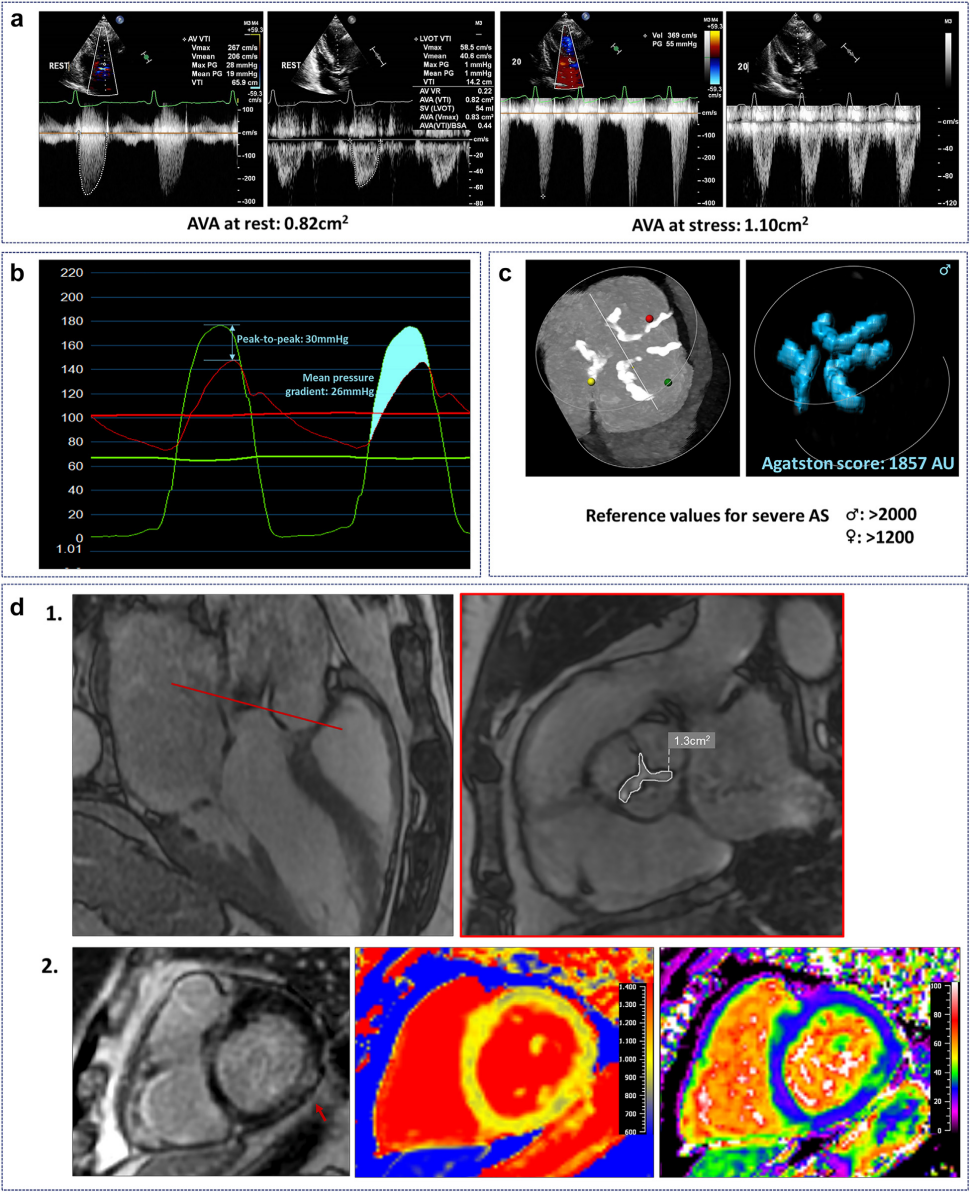
**Imaging modalities in the diagnosis and evaluation of moderate aortic stenosis and associated left ventricular changes.** (a) dobutamine stress echocardiography. Low peak aortic valve velocity and mean pressure gradient at rest in combination with aortic valve area <1.0 cm^2^ indicate low-flow low-gradient aortic stenosis. Dobutamine stress increases aortic valve area >1.0 cm^2^, indicating pseudo-severe, or moderate, aortic stenosis. (b) Aortic valve calcification in a male patient showing Agatston score of 1857 arbitrary units, corresponding to moderate aortic stenosis. (c) invasive pressure measurements showing measurements fitting with moderate aortic stenosis. (d.1) left: systolic cine image in 3-chamber view; and right: short axis aortic valve with aortic valve area measurement at level of red line in left. (d.2) left: late gadolinium enhancement image of the basal short axis with mid-wal enhancement inferolateral (red arrow). Middle: short axis T1 map. Right: extracellular volume map at the level of the papillary muscles with normal native T1 and extracellular volume in the septum and increased T1 and extracellular volume in the area with late gadolinium enhancement. Abbreviations: AS, aortic stenosis; AU, Agatston units; AVA, aortic valve area.

With dobutamine stress echocardiography (Figure 1a), the aim is to rectify impaired flow and enhance interpretation of discordant echo Doppler findings through the positive inotropic effects of dobutamine. Calculated AVA at peak stress levels (usually at 20 μg dobutamine per kg body weight per minute) separates pseudo-severe or moderate AS (AVA ≥1.0 cm^2^) from true severe AS (AVA remains <1.0 cm^2^). Studies confirmed that patients with true severe AS have a worse prognosis than patients with pseudo-severe/moderate AS and benefit from aortic valve replacement.^10^ Another modified echocardiography-derived method to solve discordant Doppler findings is to measure the projected AVA assuming that the flow rate would be corrected to 250 mL/s. This projected AVA improved diagnostic accuracy in the Truly or Pseudo-Severe Aortic Stenosis study.^11^^,^^12^ A projected AVA ≤1.2 cm^2^ seemed associated with more impaired survival.^13^

Importantly, discordance in gradient measurements may not be caused solely by low transvalvular flow. Echocardiography as a modality holds intrinsic limitations when compared to invasive transvalvular gradient measurement (Figure 1b). A key difference is that invasive measurements register actual pressures, while echocardiography relies on measured velocities and mathematical conversions using the simplified Bernoulli equation. Recent studies describe a discordance between echocardiography and left-heart catheterization when it comes to determination of aortic valve gradients.^14^^,^^15^ Although this discordance is most pronounced after transcatheter aortic valve replacement (TAVR), it is also observed in native aortic valve stenosis.^14^^,^^15^ Several explanations have been proposed to interpret the observed discrepancies. First, echocardiography relies on the use of the simplified Bernoulli equation, in which a pressure gradient is calculated from a measured velocity. The Bernoulli equation is dependent on several assumptions, which do not always hold true in the case of AS. For instance, this formula was designed for use in the presence of laminar flow with a single level of stenosis, while severe aortic valve stenosis may contain multiple levels of stenosis including aortic root calcification. Additionally, the formula does not account for the contribution of viscous forces, proximal left ventricle (LV) velocity and importantly, pressure recovery.^16^

Pressure recovery refers to the phenomenon that blood flow, after reaching its maximum velocity at the narrowest point (vena contracta), slows down in the ascending aorta.^17^^,^^18^ The velocity loss occurs when kinetic energy is converted back into potential energy (e.g. blood pressure).^19^, ^20^, ^21^ Since the invasive pressure measurement takes place above the valve (where pressure recovery has already occurred), it will be lower than the echo-derived measurement (where the cursor is manually placed over the vena contracta to measure the maximum velocity). Each of the aforementioned factors may contribute to the discordance between modalities, but it has not been established to what extent each factor contributes individually.

Nevertheless, despite aforementioned limitations in gradient determination, the value of echocardiography is indispensable for AS diagnosis because of several advantages. Echocardiography is noninvasive, readily available, has limited costs, and provides additional information which are also of importance in the workup of AS, such as insights in LVEF and cardiac remodeling.

Computed tomography can be used to quantify the amount of aortic valve calcification (AVC). Computed tomography–derived AVC (Figure 1c) correlated well with calcium volume as assessed by surgical inspection.^22^ There was good correlation between AVC and AS severity as assessed by transthoracic echocardiography. An AVC threshold of >2000 arbitrary units for men and >1200 arbitrary units for women was associated with a high likelihood of severe AS.^9^^,^^22^, ^23^, ^24^ Importantly, these thresholds were validated in AS patients with concordant Doppler findings and have not been properly validated in patients with depressed LV systolic function, low transvalvular flow and discordant Doppler findings. Nevertheless, high AVC seems associated with impaired survival and major cardiac events.^25^ At this point, AVC cannot replace dobutamine stress echocardiography to resolve discordant echocardiography Doppler findings but may be integrated in a multimodality approach to help ascertain AS severity in selected patients.

Transoesophageal echocardiography may provide a superior view for planimetric AVA measurement in the setting of poor acoustic windows with transthoracic echocardiography (TTE), for example in the presence of obesity, chest deformity, emphysema, or breast implants. Also computed tomography and cardiac magnetic resonance (CMR)^26^ are used for direct planimetric measurement of aortic valve orifice.^27^, ^28^, ^29^ However, AVA measured by computed tomography is generally larger and it has been suggested to raise cutoff thresholds for this modality.^28^ Additionally, a hybrid method can be utilized that includes LV outflow tract area measurement by computed tomography in combination with echocardiographic velocities in the simplified Bernoulli equation.^27^^,^^28^ The clinical value of this hybrid approach is unclear, and higher cutoff values have been proposed (i.e. AVA <1.2 for severe AS).^28^^,^^30^

CMR can estimate transvalvular velocities by using velocity-encoded techniques.^26^ However, CMR has lower temporal and spatial resolution than TTE and tends to underestimate peak velocities. For both TTE and CMR it can be difficult to get perpendicular to the actual flow, which will result in underestimation of the maximum velocity. Four-dimensional flow CMR (time-resolved 3D phase-contrast CMR) may enhance accuracy of flow measurements and compare better with TTE.^26^^,^^31^, ^32^, ^33^

## Moderate AS Risk Assessment

### Reduced LVEF

Multiple studies have linked moderate AS to poor clinical outcome.^3^^,^^34^ This may be amplified in patients with impaired LVEF. Low LVEF is associated with reduced survival and concomitant moderate AS may further increase the risk of death and hospitalizations (hazard ratio (HR) 2.98; 95% confidence interval (CI): 2.08 to 4.31).^35^ Observational data suggest that within a 4-year period, nearly half of patients with impaired LVEF and moderate AS will experience heart failure hospitalization or death.^34^ Earlier aortic valve replacement may improve outcomes.^35^ Another study found similar rates of all-cause mortality and heart failure hospitalization in patients with low LVEF and moderate or severe AS (62.0% vs. 62.7% during a follow-up of 3.1 years, *p* = 0.68).^36^

Afterload mismatch in patients with heart failure with reduced ejection fraction (HFrEF) is inflated by concomitant moderate AS. The valvulo-arterial impedance reflects the total afterload of the ventricle and consists of an arterial and valvular component. The latter is enhanced with moderate AS. The mainstay of guideline directed heart failure medical therapy revolves around afterload reduction (i.e., renin-angiotensin-system inhibitors, vasodilators, aldosterone antagonists, beta blockers and diuretics). High valvulo-arterial impedance is associated with increased mortality regardless of which component (i.e., the arterial or the valvular) dominates.^37^^,^^38^ Increased arterial stiffness and irreversible loss in compliance may make HFrEF patients less susceptible to vasodilators and other afterload reducing agents. In patients with concomitant moderate AS, the valvular component may become a reasonable treatment target for minimally invasive TAVR.^37^^,^^38^ This is the rationale of the TAVR to UNload the Left Ventricle in Patients With ADvanced Heart Failure trial (TAVR UNLOAD; clinicaltrials.gov identifier: NCT02661451).^39^

### Other Signs of Cardiac Damage Irrespective of LVEF

Recent studies suggested detrimental effects of moderate AS in selected patients who exhibit any degree of cardiac damage. Cardiac damage can manifest itself by morphologic changes (e.g. LV hypertrophy), diastolic dysfunction, atrial fibrillation, elevated biomarkers (N-terminal pro-B-type natriuretic peptide (NT-pro-BNP), B-type natriuretic peptide (BNP), troponin), mitral regurgitation, dilated left atrium and eventually also right heart changes that include tricuspid regurgitation and right ventricular dilation and dysfunction.^40^, ^41^, ^42^ In a large registry of 1931 patients with moderate AS, LV geometry was normal in only one-fifth.^40^ Concentric hypertrophy was the most common morphological change and appeared in approximately one-third of patients. Eccentric hypertrophy was found in 22% of patients and increased relative wall thickness with normal LV mass in 24%. Diastolic dysfunction affected 43% to 54% of patients with moderate AS.^43^^,^^44^ Additionally, speckle tracking global longitudinal strain studies have shown subclinical myocardial dysfunction in patients with AS even before LV systolic dysfunction occurs.^45^ Impaired strain is most evident with severe AS, but also features in moderate and even mild AS.^45^

These echocardiographic parameters have been consistently linked to worse survival outcomes.^40^^,^^41^^,^^43^^,^^44^^,^^46^, ^47^, ^48^ Particularly an elevated E/e’ ratio and impaired LV global longitudinal strain seem independently associated with increased mortality regardless of symptoms or impaired LV function.^48^ Right heart involvement characterized by 1) pulmonary artery pressure >60 mmHg, 2) moderate or more than moderate tricuspid regurgitation, or 3) tricuspid annular plane systolic excursion <16 mm are indicative for poor clinical outcome with >60% 5-year incidence of all-cause mortality, stroke, heart failure hospitalization, or myocardial infarction.^42^

Inappropriate loading conditions such as AS may ignite the process of myocardial fibrosis that can be detected by CMR (Figure 1d).^49^ Two phenotypes of fibrosis may be recognized. Reactive interstitial fibrosis has a diffuse pattern with concomitant extracellular space expansion that emerges early in the disease progress and is potentially reversible.^50^^,^^51^ Native T1 values and extracellular volume fractions are CMR parameters that may accompany this type of fibrosis and have been shown to correlate with AS severity.^52^, ^53^, ^54^, ^55^ Extracellular volume fraction indexed to body surface area has shown good correlation to degree of fibrosis in patients across the AS severity spectrum.^52^

Replacement fibrosis in the context of moderate AS is often characterized by irreversible midwall late gadolinium enhancement on CMR, and signals more advanced disease and is more frequent with moderate (31%) and severe AS (32%) than with mild AS (5.4%).^51^^,^^52^

The type of LV fibrosis pattern may have prognostic implications in the context of moderate and severe AS.^52^^,^^56^, ^57^, ^58^ In patients with severe AS, extracellular volume fraction appeared a stronger predictor for mortality than LVEF.^54^ Replacement fibrosis was a strong predictor for mortality regardless of aortic valve intervention or stage of AS (i.e., moderate or severe AS).^59^ Interestingly, in patients with AS and replacement fibrosis or midwall late gadolinium enhancement, aortic valve intervention during follow-up improved survival.^56^

Various biomarkers are linked to impaired outcome in the context of AS.^55^^,^^60^ NT-pro-BNP is a biomarker of LV wall stress and correlates with onset and severity of symptoms and perioperative and long-term mortality.^60^^,^^61^ In a retrospective study of 261 patients with moderate AS, elevated NT-pro-BNP was an independent predictor for mortality.^62^ Troponin correlated with LV mass index (r = 0.50, *p* < 0.001), peak aortic jet velocity (r = -0.32, *p* < 0.001) and midwall late gadolinium enhancement pattern fibrosis.^55^ Also, elevated troponin levels were an independent predictor for mortality and need for aortic valve replacement.^55^

**Figure 2:**
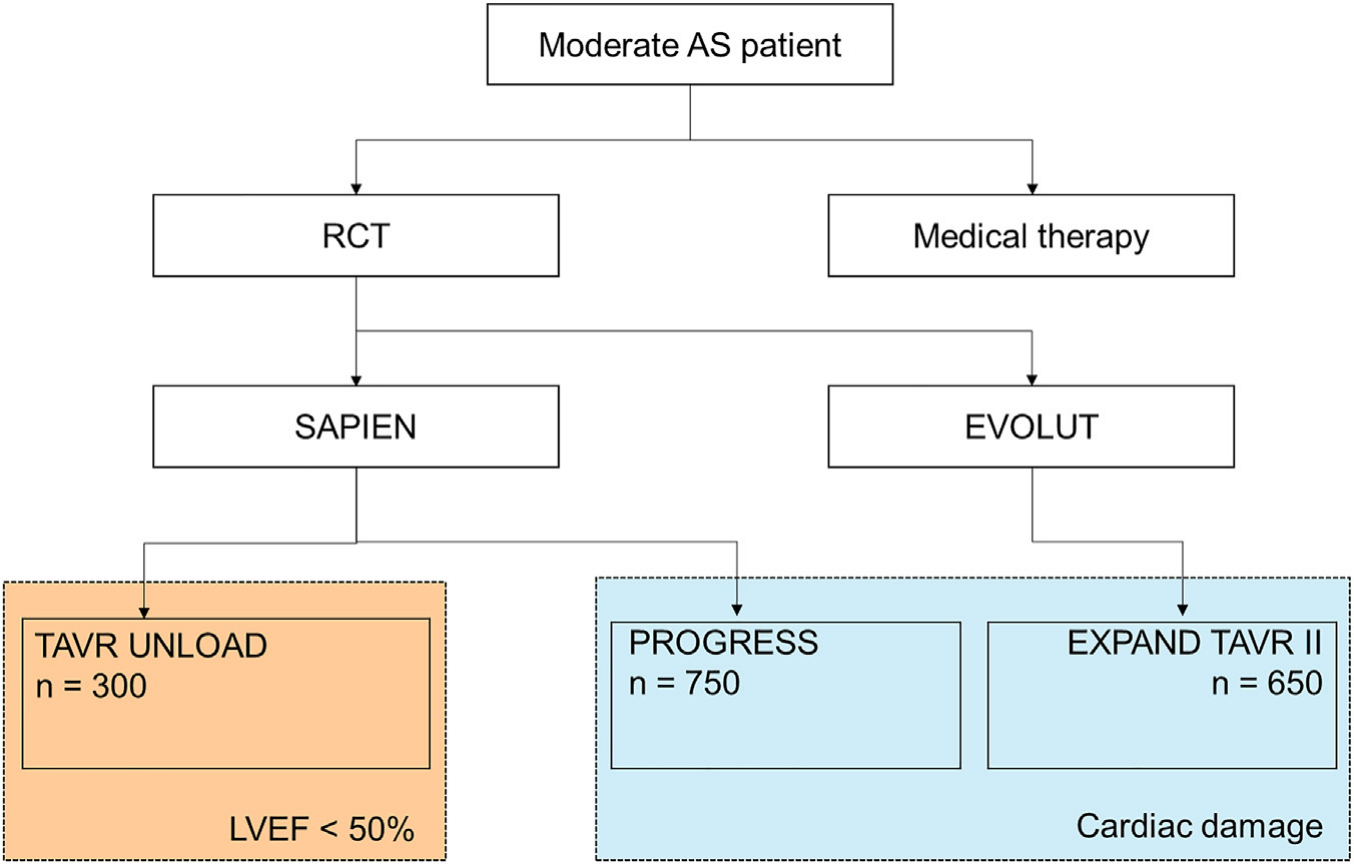
**Ongoing trials on transcatheter aortic valve replacement for moderate aortic stenosis**. Abbreviations: AS, aortic stenosis; LVEF, left ventricular ejection fraction; RCT, randomized controlled trial; TAVR, transcatheter aortic valve replacement; TAVR UNLOAD, Transcatheter Aortic Valve Replacement to UNload the Left Ventricle in Patients With ADvanced Heart Failure trial.

Two randomized controlled trials are investigating TAVR in patients with moderate AS and cardiac damage independent of LVEF: the Prospective, Randomized, Controlled Trial to Assess the Management of Moderate Aortic Stenosis by Clinical Surveillance or Transcatheter Aortic Valve Replacement (PROGRESS; clinicaltrials.gov identifier: NCT04889872) and the Evolut EXPAND TAVR II (clinicaltrials.gov identifier: NCT05149755) trials (Figure 2).

## Treatment of Moderate AS

Current cardiology society guidelines do not recommend aortic valve replacement for moderate AS, except in the context of concomitant valve or coronary surgery.^6^^,^^7^

Various strategies have been studied to affect the progression from moderate to severe AS. For now, clinical care of moderate AS revolves around guideline directed treatment of heart failure, hypertension, and coronary artery disease.

### Heart Failure Therapy

Guideline-derived optimal medical therapy for heart failure should also be applied to patients with HFrEF and moderate AS. Betablockers, renin-angiotensin-system inhibitors (preferably with neprilysin inhibition), mineralocorticoid receptor antagonists and sodium-glucose transport protein 2 (SGLT2) inhibitors need to be titrated to maximally tolerated doses.^63^^,^^64^ Furthermore, adequate rate or rhythm control in the presence of atrial fibrillation and cardiac resynchronization therapy with left bundle branch block are strongly recommended. The role for complete revascularization of concomitant coronary artery disease seems clinically sound but was recently challenged by a randomized trial.^65^ Diuretics may be required to correct congestion. Apart from these general heart failure considerations, no dedicated randomized controlled trials have been concluded to help formulate strong guideline recommendations for HFrEF and moderate AS.

Recent trials revealed consistent clinical benefits with SGLT2 inhibitors in patients with heart failure with preserved ejection fraction or heart failure with moderate-reduced ejection fraction.^66^^,^^67^ SGLT2 inhibitors seem an intriguing strategy for patients with moderate AS and preserved LVEF. However, patients with AS were excluded from the landmark SGLT2 inhibitor trials. Of note, the dapagliflozin after transcatheter aortic valve implantation trial (DapaTAVI; clinicaltrials.gov identifier: NCT04696185)^68^ will investigate the SGLT2 inhibitor dapagliflozin in patients after successful TAVR.

### Strategies to Affect AS Progression

Statins were tested to affect AS progression in patients with moderate AS.^69^, ^70^, ^71^ In the Simvastatin and Ezetimibe in Aortic Stenosis (SEAS) study,^70^ 1873 patients with asymptomatic mild-to-moderate AS were randomized in a 1:1 ratio to receive 40 mg simvastatin plus 10 mg ezetimibe or placebo. There was no difference in the primary composite endpoint of cardiovascular death, aortic valve replacement, nonfatal myocardial infarction, hospitalization for unstable angina pectoris, heart failure, coronary artery bypass grafting, percutaneous coronary intervention, and nonhemorrhagic stroke (HR 0.96, 95% CI 0.83 to 1.12, *p* = 0.59) and AS progression was similar (0.61 ± 0.59 m/s vs 0.62 ± 0.61 m/s for treatment group and placebo respectively; *p* = 0.83).

More recently, elevated levels of lipoprotein (a) and oxidized phospolipids have been linked to disease progression of calcific AS and a connection has been found with a gene variant coding for lipoprotein (a) expression.^72^^,^^73^ Proprotein convertase subtilisin/kexin type 9 inhibiting drugs such as alirocumab and evolocumab can achieve 15% to 30% reductions in lipoprotein(a) levels^74^, ^75^, ^76^ and as such, may offer an alternative treatment strategy in the medical monitoring of moderate AS patients. An exploratory analysis of the ‘Further Cardiovascular Outcomes Research with proprotein convertase subtilisin/kexin type 9 Inhibition in Subjects with Elevated Risk’ (FOURIER) trial,^75^ which randomized 27,564 patients at increased risk of cardiovascular events already taking statin therapy to evolocumab or placebo, found a numerically lower incidence of AS events (defined as new or worsening AS or aortic valve replacement) in the treatment arm compared to the placebo arm (0.27% [95% CI: 0.17% to 0.44%] vs 0.41% [95% CI: 0.28% to 0.59%]) coupled with a 26.9% reduction in lipoprotein (a) levels.^77^

### Aortic Valve Intervention

So far, no randomized controlled trials have established the value of surgical aortic valve replacement or TAVR in patients with moderate AS. TAVR UNLOAD completed the enrollment of 178 patients in February 2023 and primary outcome data should be available in Q3 2024. PROGRESS is expected to wrap up its enrollment in December 2023 with outcome data expected in 2026. Patient inclusion in the EXPAND TAVR II pivotal trial is ongoing.

Nonetheless, observational studies have been published that hinted towards clinical benefits and improved 5-year survival with aortic valve replacement in patients with moderate AS who underwent coronary artery bypass grafting (HR 0.29, 95% CI: 0.20 to 0.44 when AS is determined by mean gradient; HR 0.37, 95% CI: 0.20 to 0.67 when AS is determined by AVA).^78^

A retrospective propensity matched analysis of HFrEF patients with or without moderate AS reported worse outcome in the presence of moderate AS and clinical benefits of aortic valve replacement (HR: 0.59; 95% CI: 0.65 to 0.98, *p* = 0.04).^35^ In patients with preserved LVEF and moderate AS, aortic valve replacement was also associated with improved survival.^4^

**Figure 3:**
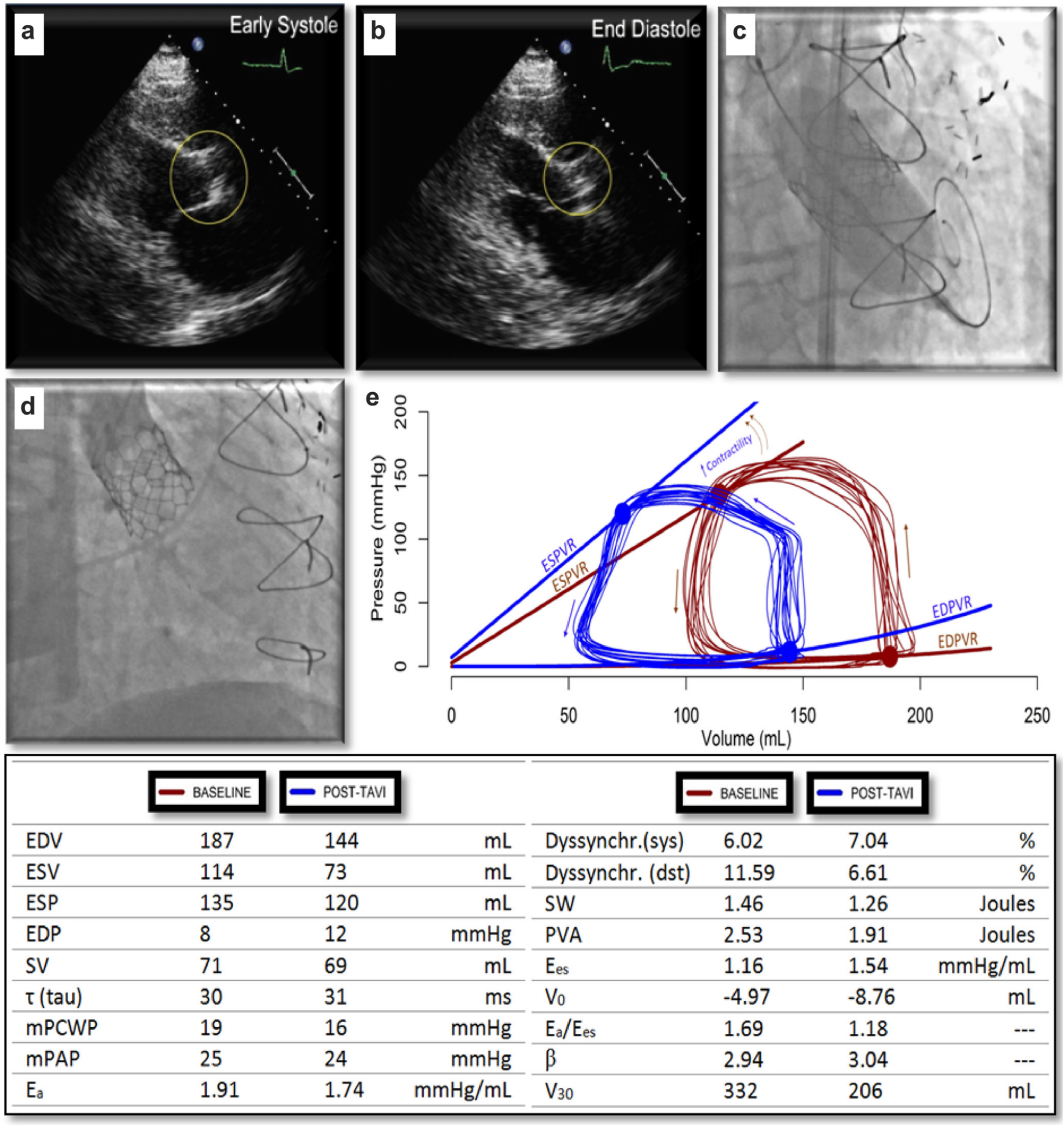
**Pressure-volume analysis before and after transcatheter aortic valve replacement in moderate aortic stenosis.** (Panel a and b) illustrating moderate aortic stenosis with aortic valve area of 1.1 cm^2^. (Panel c and d) uneventful transcatheter aortic valve replacement. (Panel e) left-shift of pressure-volume loops with decrease in end-diastolic and end-systolic volumes (unloading) and improved contractility. Abbreviations: E_a_, arterial elastance; EDP, end-diastolic pressure; EDPVR, end-diastolic pressure-volume relationship; EDV, end-diastolic volume; E_es_, end-systolic elastance; ESP, end-systolic pressure; ESPVR, end-systolic pressure-volume relationship; ESV, end-systolic volume; mPAP, mean pulmonary artery pressure; mPCWP, mean pulmonary capillary wedge pressure; PVA, pressure-volume area; V_0_, volume at zero mmHg; V_30_, volume at 30mmHg diastolic pressure; SV, stroke volume; SW, stroke work; TAVR, transcatheter aortic valve replacement.

An invasive left ventricular pressure volume relationship study demonstrated immediate mechanical unloading and segmental resynchronization following (transcatheter) aortic valve replacement in a patient with HFrEF and moderate AS, which underpins the pathophysiological rationale for aortic valve replacement in the setting of moderate AS (Figure 3).^79^ Moreover, a recent propensity matched study analysis suggested superiority in terms of 2-year survival with TAVR over watchful waiting in patients with pseudo-severe (moderate) AS (overall: 65.4% vs 48.8%, *p* = 0.0002; cardiovascular 80.4% vs 58.5%, *p* < 0.0001).^80^

This figure was previously published in *Bastos et al. JACC: Cardiovascular Interventions, 12(7); 684-6 (2019)* and reused with permission. Copyright Elsevier.

## Conclusions

A growing body of clinical evidence links moderate AS to poor clinical outcome. Integrated multimodality imaging may be required to establish the diagnosis of moderate AS. Risk stratification models are emerging to identify moderate AS patients who may benefit from aortic valve replacement. The impact of TAVR for moderate AS is currently tested in randomized controlled trials.

## Funding

This research did not receive any specific grant from funding agencies in the public, commercial, or not-for-profit sectors.

## Disclosure Statement

Nicolas M. Van Mieghem has received grant support/research contracts from Abbott Vascular, Boston Scientific, Medtronic, Edwards Lifesciences, Daiichy Sankyo, Astra Zeneca and PulseCath BV and has received consulting/speaker fees from Abbot Vascular, Boston Scientific Corporation, Medtronic, Daiichy Sankyo, PulseCath BV, JenaValve, and Amgen. Ricardo P.J. Budde has received institutional support and speaker fees from Siemens and is member of the executive board of the European Society of Cardiovascular Radiology. Alexander Hirsch has received a research grant and consultancy fees from GE Healthcare and speaker fees from GE Healthcare and Bayer. He is also a member of the medical advisory board of Medis Medical Imaging Systems. All other authors declare to have no conflicts of interest.
